# Supplementary material for: Energy losses in photovoltaic generators due to wind patterns
Source: Commun Eng. 2023 Sep 22;2:66. doi: 10.1038/s44172-023-00119-7 (PMC10956078; doi:10.1038/s44172-023-00119-7)
Supplement: Supplementary file 2 — Supplementary Figures [file 44172_2023_119_MOESM2_ESM.pdf]

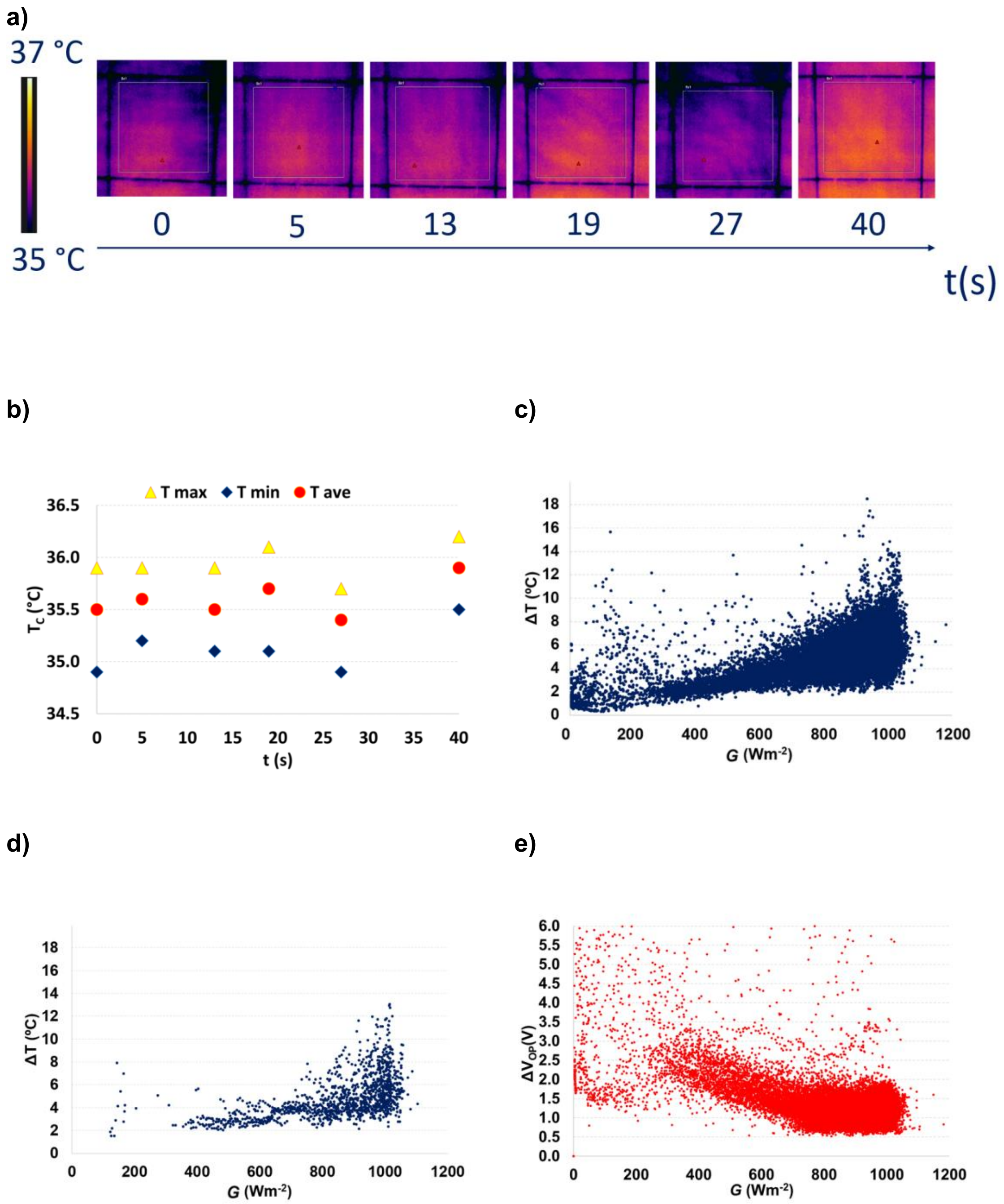

Fig. S1 Temperature and operating voltage differences. **(a)** Sequence of six thermography centred in a PV cell currently operating outdoors, taken during 40 seconds. The red and blue triangles in each thermography represents the maximum and minimum temperatures, respectively. Wind speed during the measurements was less than 2 ms^{-1}, with slight variations (wind gusts) corresponding to the 0s and 27s. **(b)** Maximum, minimum and average temperatures measured with the thermography camera. **(c)** Temperature difference ΔT vs in-plane irradiance measured between February 27th 2017 and July 20th 2020. **(d)** Temperature difference ΔT vs in-plane irradiance measured between June 19th and July 3rd of 2018. **(e)** Operating voltage vs in-plane irradiance measured simultaneously with ΔT in c).

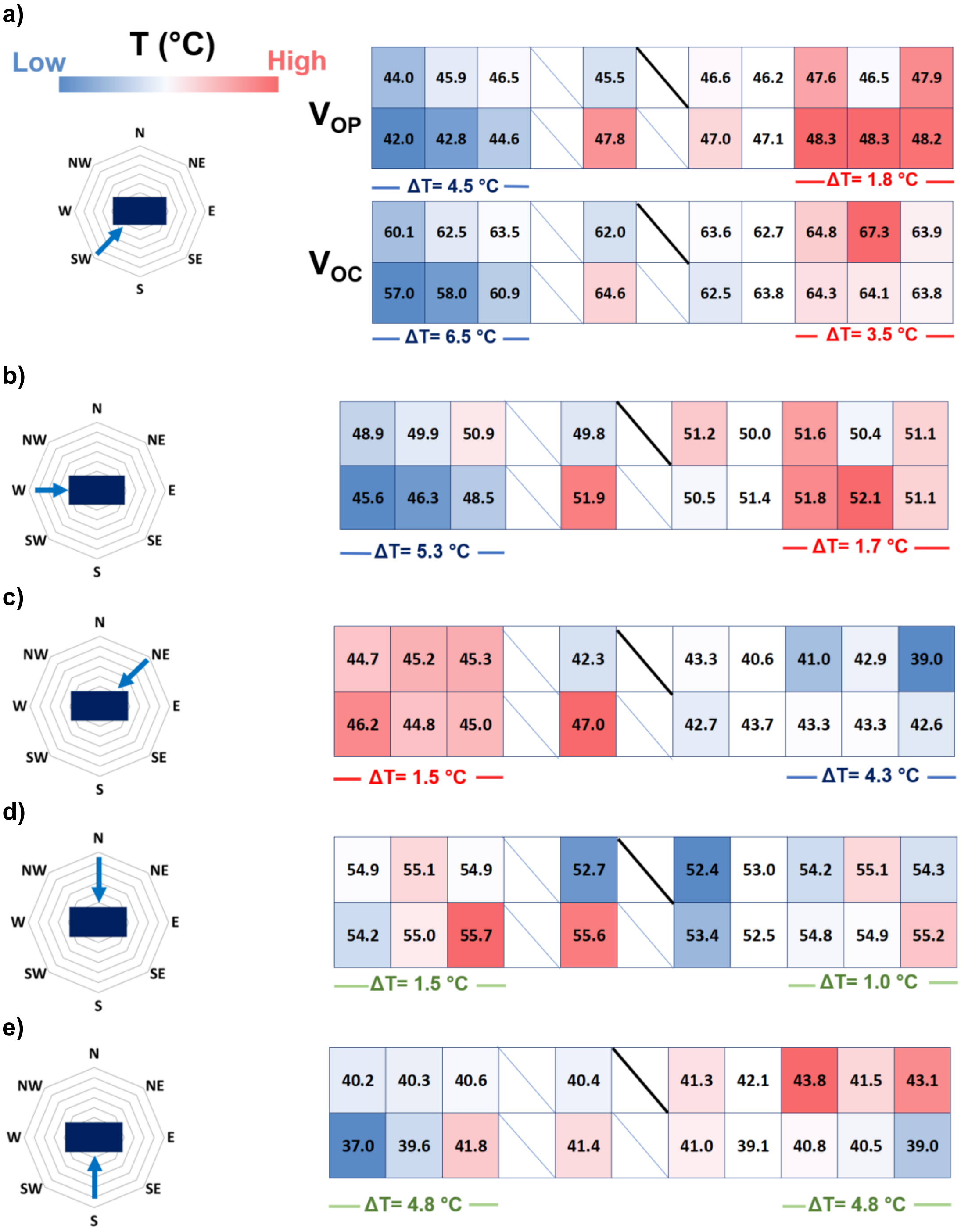

Fig. S2 Temperature differences with different wind incidences. Front view of the PV generator with the individual temperatures of 18 PV modules, recorded in a single moment. **(a)** South-West quadrant incidence in two distinct days, with the PV generator delivering energy into the grid and in open-circuit. Wind speed in both cases is v_∞= 6.2 ms^{-1}, with G ≥ 960 Wm^{-2}. **(b)** West quadrant incidence, parallel to the PV panel. **(c)** Diagonal rear incidence, from the North-East quadrant. **(d)** Rear wind incidence, from the North quadrant. **(e)** Frontal wind incidence, from the South quadrant. In each wind rose, N, NE, E, SE, S, SW, W and NW represent the North, North-East, East, South-East, South, South-West, West and North-West, respectively.

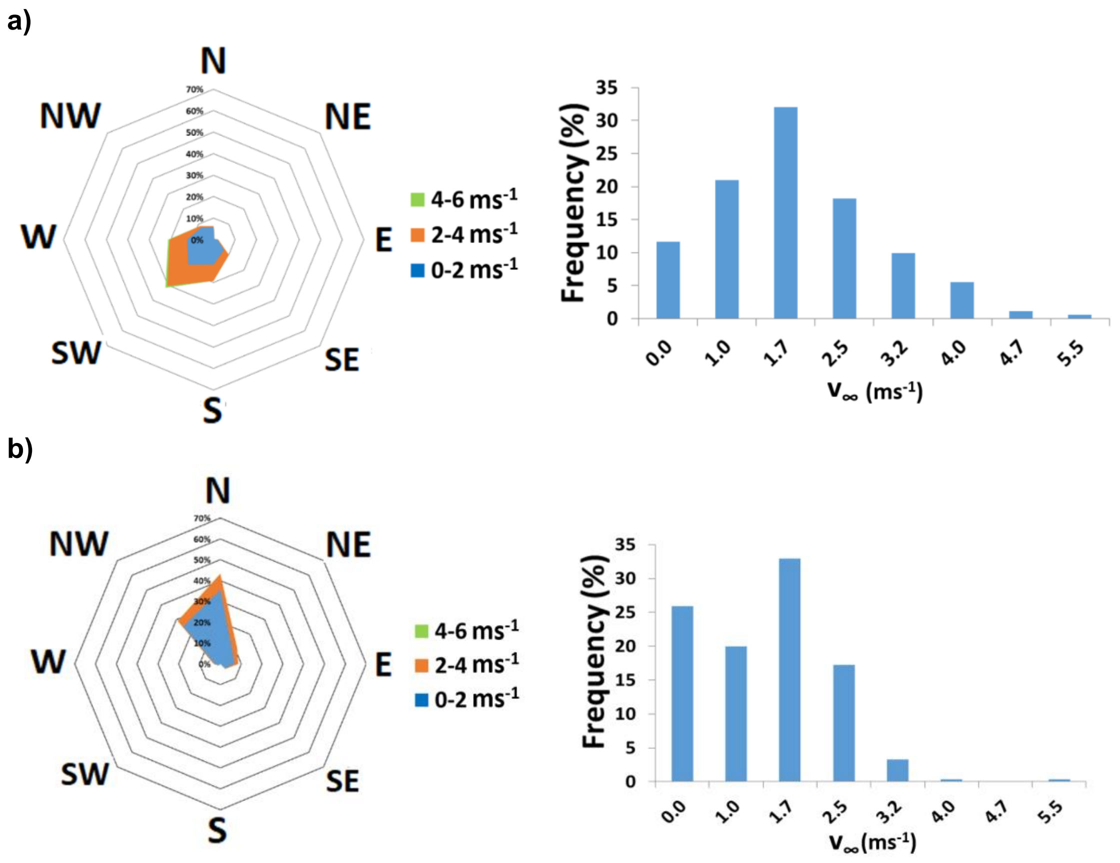

Fig. S3 Variations with the wind patterns, for low wind speeds. (a) frontal wind incidence, MML_{DAY}=0.25%. **(b)** rear wind incidences, MML_{DAY}=0.17%. In each wind rose, N, NE, E, SE, S, SW, W and NW represent the North, North-East, East, South-East, South, South-West, West and North-West, respectively.

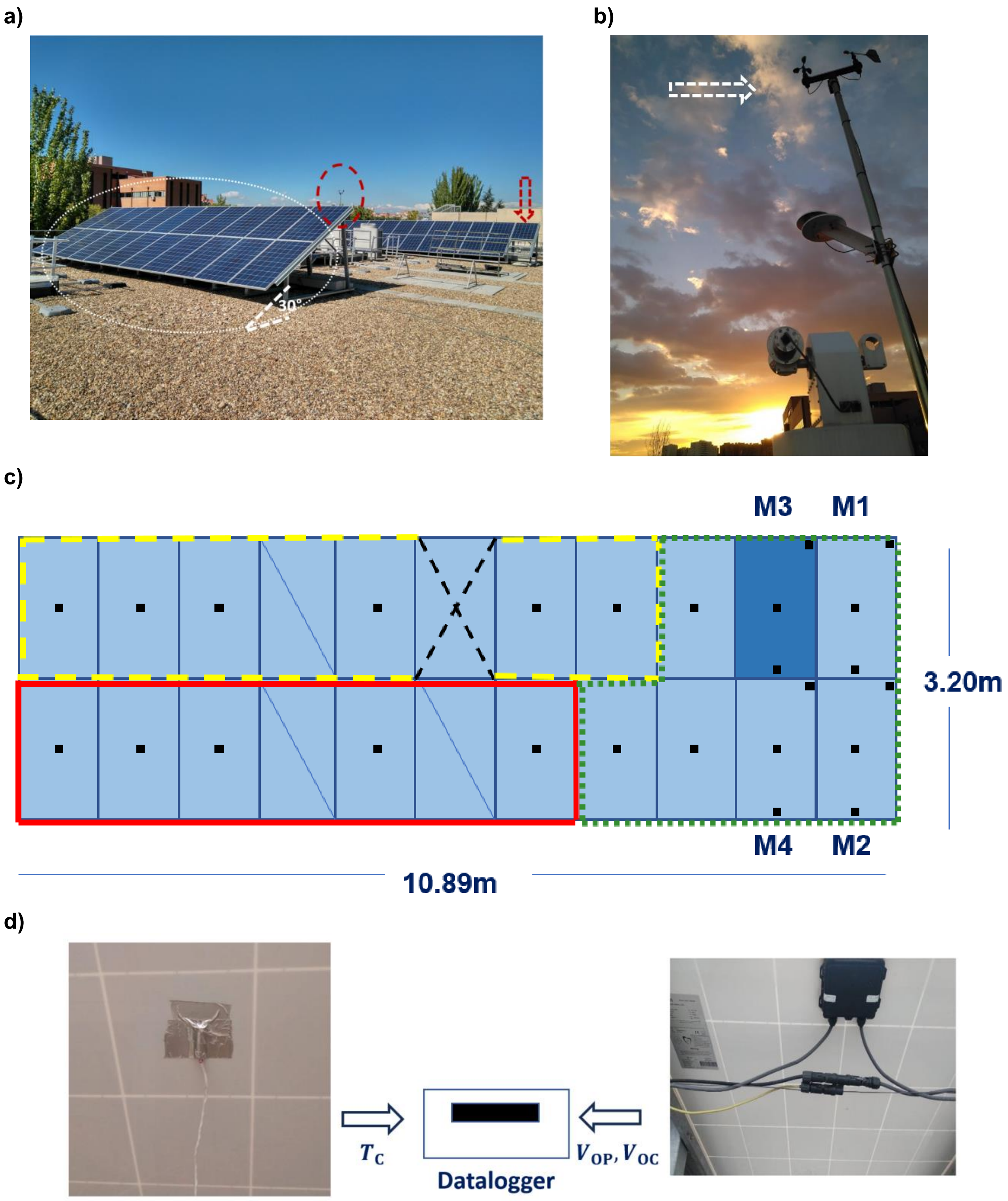

Fig. S4 Experimental setup. **(a)** IES-UPM terrace with two monofacial PV generators. The white dashed ellipse marks the analysed PV generator. The red dashed ellipse marks the location of the anemometer – detailed with the white dashed arrow in **(b)**. The small red dashed arrow identifies the calibrated module measuring both T_C and G_{EF}. **(c)** Front view scheme of the PV generator (inside white dashed ellipse in (a), showing the location of all the PT1000 sensors – detailed in d) - distributed in it. The modules without PT1000 sensors are marked with the diagonal blue line. The two diagonal dashed black lines mark the disconnected module. The module M3, with a distinct colour, is the calibrated module used for the thermal drop experiment. The three strings with 7 modules connected in series each are distinguished with a red continuous line, yellow dashed line and green dashed line. **(d)** PT 1000 sensor and T shape connector.
