# Supplementary material for: Energy losses in photovoltaic generators due to wind patterns
Source: Commun Eng. 2023 Sep 22;2:66. doi: 10.1038/s44172-023-00119-7 (PMC10956078; doi:10.1038/s44172-023-00119-7)
Supplement: Supplementary file 3 — Reporting Summary [file 44172_2023_119_MOESM3_ESM.pdf]

Corresponding author(s): DAPR COMMSENG-23-0092B

Solar Cells Reporting Summary

Nature Portfolio wishes to improve the reproducibility of the work that we publish. This form is intended for publication with all accepted papers reporting the characterization of photovoltaic devices and provides structure for consistency and transparency in reporting. Some list items might not apply to an individual manuscript, but all fields must be completed for clarity.

For further information on Nature Research policies, including our data availability policy, see Authors & Referees.

Experimental design

Please check the following details are reported in the manuscript, and provide a brief description or explanation where applicable.

1. Dimensions

Area of the tested solar cells

- [ ] Yes — *Report the area of the tested solar cells (e.g. aperture area, active area).*
- [x] No — The dimensions of the cell (i.e. just one cell) are not relevant for the present purposes.

Method used to determine the device area

- [x] Yes — As this is a study of a PV generator, the relevant information is the PV module area. So, this data is provided by the datasheet.
- [ ] No — *Explain why this information is not reported/not relevant.*

2. Current-voltage characterization

Current density-voltage (J-V) plots in both forward and backward direction

- [ ] Yes — Current density was not analysed in this paper.
- [x] No

Voltage scan conditions

- [ ] Yes — *Provide a description of the measurement conditions (e.g. scan direction, speed, dwell times).*
- [x] No — Voltage scan for cell analysis purposes was not realised in this paper.

Test environment

- [x] Yes — Typical environmental conditions for the location during the whole year, as the PV generator is located on a terrace, considering the period free of shades in the PV generator and the irradiance greater than 700 Wm-2.
- [ ] No — *Explain why this information is not reported/not relevant.*

Protocol for preconditioning of the device before its characterization

- [ ] Yes — *Provide a description of the protocol.*
- [x] No — The main objective is the analysis in real conditions. Preconditioning does not apply here.

Stability of the J-V characteristic

- [ ] Yes — *Provide a description of the method used. The stability of the J-V characteristic can be verified with time evolution of the maximum power point or with the photocurrent at maximum power point; see ref. 5 for details.*
- [x] No — Current density was not analysed in this paper.

3. Hysteresis or any other unusual behaviour

Description of the unusual behaviour observed during the characterization

- [ ] Yes — *Provide a description of hysteresis or any other unusual behaviour observed during the characterization.*
- [x] No — None of the related phenomena is observed in this kind of analysis.

Related experimental data

- [ ] Yes — *Provide a description of the related experimental data.*
- [x] No — There is no related experimental data concerning PV cell phenomena.

4. Efficiency

External quantum efficiency (EQE) or incident photons to current efficiency (IPCE)

- [ ] Yes — *Provide a description of the technique used.*
- [x] No — EQE or IPCE were not analysed in this paper.

A comparison between the integrated response under the standard reference spectrum and the response measure under the simulator

☐ Yes
☒ No

State where this information can be found in the text.

Does not apply to this study. Analysis performed in a PV generator exposed to the natural solar spectrum.

For tandem solar cells, the bias illumination and bias voltage used for each subcell

☐ Yes
☒ No

Provide a description of the measurement conditions.

Does not apply in this study.

5. Calibration

Light source and reference cell or sensor used for the characterization

☒ Yes
☐ No

The reference PV module is from the same manufacturer of the modules from the PV generator. Thus, any uncertainty regarding the spectral response for distinct devices, at least for real applications, is disregarded. This ensures that the irradiance measured by the reference module is the same irradiance reaching the PV generator (reference module installed in-plane with the PV generator).

Explain why this information is not reported/not relevant.

Confirmation that the reference cell was calibrated and certified

☐ Yes
☒ No

Identify the independent certification laboratory.

The PV module itself is not certified. However, all the calibrations procedures are made with a calibrated cell previously calibrated with a primary cell. These details can be find in the related references in the paper (Carrillo 2017).

Calculation of spectral mismatch between the reference cell and the devices under test

☐ Yes
☒ No

Provide a value of the spectral mismatch and/or a description of how it has been taken into account in the measurements.

This calculation is not necessary, as the reference module and the modules of the PV generator are made by the same manufacturer. For practical purposes, this is disregarded.

6. Mask/aperture

Size of the mask/aperture used during testing

☐ Yes
☒ No

Report the size of the mask/aperture.

Does not apply in this study.

Variation of the measured short-circuit current density with the mask/aperture area

☐ Yes
☒ No

Report the difference in the short-circuit current density values measured with the mask and aperture area.

Does not apply in this study.

7. Performance certification

Identity of the independent certification laboratory that confirmed the photovoltaic performance

☐ Yes
☒ No

Identify the independent certification laboratory.

Does not apply directly in this study. Related studies, cited in the paper, can provide these informations with more accuracy (Carrillo 2017).

A copy of any certificate(s)

☐ Yes
☒ No

Certificate copies should be provided in the Supplementary information. Please state the supplementary item number.

Certification does not apply for this study.

8. Statistics

Number of solar cells tested

☐ Yes
☒ No

Report how many solar cells have been tested, specifying the number of individual substrates.

Apart from the PV generator, solar cells were not tested.

Statistical analysis of the device performance

☐ Yes
☒ No

State where this information can be found in the text.

Does not apply here.

9. Long-term stability analysis

Type of analysis, bias conditions and environmental conditions

☐ Yes
☒ No

Provide a description of the type of analysis, bias conditions and environmental conditions (e.g. illumination type, temperature, atmosphere humidity, encapsulation method, preconditioning temperature, bias) for each long-term stability analysis carried out; see ref. 7 and 8 for details.

Does not apply, in these lines, for the present purposes.
